# Effectiveness of Topiramate Versus Acetazolamide in the Management of Idiopathic Intracranial Hypertension: ASystematic Review and Meta-Analysis

**DOI:** 10.3390/medicina61030450

**Published:** 2025-03-04

**Authors:** Abdullah Almaqhawi, Alia Alokley, Reham Alamri, Razan Alabdulqader, Ahmad Alali, Ayat Aleid, Amani Alhejji, Maryam N. ALNasser

**Affiliations:** 1 Department of Family and Community Medicine, College of Medicine, King Faisal University, Al Hofuf P.O. Box 400, Saudi Arabia; 2 Departments of Clinical Neurosciences, College of Medicine, King Faisal University, Al Hofuf P.O. Box 400, Saudi Arabia; aalokley@kfu.edu.sa; 3 College of Medicine, King Faisal University, Al Hofuf P.O. Box 400, Saudi Arabia; rehamalamri211@gmail.com (R.A.); rzanalabdulgader@gmail.com (R.A.); 220007941@student.kfu.edu.sa (A.A.); ayatt.jassim@gmail.com (A.A.); amanihejji@gmail.com (A.A.); 4 Department of Biological Sciences, College of Science, King Faisal University, Al Hofuf P.O. Box 400, Saudi Arabia; malnasser@kfu.edu.sa

**Keywords:** idiopathic intracranial hemorrhage, primary pseudotumor cerebri syndrome, acetazolamide, topiramate, intracranial pressure

## Abstract

*Background and Objectives*: Primary pseudotumor cerebri syndrome, another name for idiopathic intracranial hypertension (IIH), is a neurological condition marked by elevated intracranial pressure (ICP) that can result in papilledema without a known etiology. The purpose of this study is to compare the efficacy of topiramate and acetazolamide as medical treatments for IIH and to evaluate the long-term outcomes of both medications. *Materials and Methods*: This systematic review and meta-analysis followed the PRISMA guidelines and was approved by the International Prospective Register for Systematic Reviews (PROSPERO). This study included randomized clinical trials, retrospective and prospective cohort studies, and patients with idiopathic intracranial hypertension (IIH). Data extraction was performed using the Rayyan application, and the risk of bias was assessed using the Critical Appraisal Skills Program (CASP). *Results*: The findings revealed a statistically significant 67% increase in the likelihood of improvement at 6 months compared to the baseline with the administration of acetazolamide and topiramate. After six months of the drug administration, there was a 3.6 times decrease in visual obscuration compared to the baseline. A significant advantage of topiramate in IIH is the added benefit of weight loss, since obesity is a modifiable risk factor. However, acetazolamide remains the conventional treatment. *Conclusions*: This study found that acetazolamide and topiramate are both effective therapies for idiopathic intracranial hypertension (IIH), improving visual metrics and decreasing cerebrospinal fluid pressure. Topiramate aids in weight reduction, while acetazolamide is recommended for its ability to lower CSF pressure and alleviate visual changes. A combination treatment of topiramate and acetazolamide is recommended for better results.

## 1. Introduction

Based on current statistics from a large database of 85 million individuals, the prevalence of IIH has increased dramatically during the previous decade. From 2015 to 2022, the prevalence increased by a factor of 1.35, rising from 7.3 (95% CI 6.9–7.7) individuals per 100,000 to 9.9 (95% CI 9.5–10.3) individuals per 100,000 in 2022 [1]. This highlights the growing burden of IIH and underscores the importance of effective management strategies.

As the name refers to “idiopathic”, there still lacks a full understanding of the pathogenesis of IIH. On the other hand, some theories have been proposed explaining the mechanism behind the rise in intracranial pressure, such as increased CSF production; impaired CSF absorption, which subsequently causes cerebral edema; or the outflow obstruction of dural venous sinuses. The hypersecretion of CSF is due to dysfunctional epithelial cells located on the apical surface of the choroid plexus, the upregulation of Na+/K+ATPase transporter activity, or the dysfunction of the AQP1 channel function [2].

Carbonic anhydrase is a metalloenzyme present in red blood cells, plasma, the proximal tubule of the kidney, the liver, the ciliary body of the eye, and the choroid plexus. Carbonic anhydrase equilibrates the reaction between CO_2_, bicarbonate, and protons [3]. The inhibition of CA limits the formation of bicarbonate and hydrogen ions and therefore induces diuresis [4].

The primary carbonic anhydrase inhibitors, sulfonamides and their isosteres (sulfamates/sulfamides), are known to be effective in reducing the ICP by reducing CSF production via the inhibition of the Na+/K+ATPase transporter and the downregulation of AQP1 expression. CAIs have been used for several decades in several conditions, such as anti-epileptic, anti-obesity, anti-cancer, and anti-glaucoma treatment [2,3].

The initial management of IIH is medical therapy with diuretics. However, in refractory cases, or when the symptoms of IIH worsen, such as through visual deterioration, surgical interventions must be considered. Procedures like serial lumbar puncture, optic nerve sheath fenestration, lumboperitoneal shunt, ventriculoperitoneal shunt, and venous sinus stenting are used to alleviate the symptoms of IIH [5].

The first line of treatment for IIH is acetazolamide, which is a sulfonamide carbonic anhydrase inhibitor that decreases the CSF production by the mechanism mentioned above and subsequently reduces the carbonic anhydrase inhibitors (CAIs). Acetazolamide is also used in a variety of other conditions such as epilepsy, heart failure with volume overload, acute mountain sickness, and glaucoma [6]. Another used medication with a diuretic effect that is known to be effective in IIH is topiramate, which is a sulfamate carbonic anhydrase inhibitor. Topiramate was first synthesized as part of a research project to test its ability to block gluconeogenesis. Although the topiramate was devoid of hypoglycemic activity, the structure of the 0-sulfamate moiety resembled the sulfonamide moiety in acetazolamide; therefore, topiramate offered a possible anticonvulsant therapeutic action [7].

Other diuretic medications, including furosemide, amiloride, and spironolactone, are also used to decrease the ICP [8]. Although acetazolamide is the initial treatment for IIH, few studies have examined its effect in alleviating the symptoms of IIH. A randomized clinical trial compared the efficacy between acetazolamide-plus-diet and placebo-plus-diet over a 6–12-month period with treatment exchange or discontinuation after a 6-month period. The most significant improvement was found in those who were on acetazolamide therapy for 12 months. Interestingly, the improvement in visual field MD was seen as well in those who were on placebo then no acetazolamide treatment. This finding highlights the impact of lifestyle management and weight loss in treating the symptoms of IIH [9]. The most common side effects associated with acetazolamide therapy are fatigue, the tingling of the hands and feet, dysgeusia, gastrointestinal tract symptoms such as nausea, and metabolic acidosis. Thus, the maximum dose recommended is 4 g/day as a precaution to decrease the probability of these side effects occurring [4,10]. Another randomized clinical trial examined the effect of acetazolamide (4 g/day) versus placebo on visual function with a low-sodium weight-reduction diet in both study groups. The improvement of the perimetric mean deviation and Frisén papilledema grade were largely observed in the acetazolamide group in comparison to the low sodium diet alone group at 6 months, in patients with mild vision loss [11].

Topiramate inhibits the isozymes of carbonic anhydrase enzyme at high doses (200 mg, twice daily); therefore, it could be as effective as acetazolamide in decreasing the CSF, but at much higher doses in comparison to acetazolamide [7]. One of the side effects of topiramate is weight loss [12]. Thus, it offers a great therapeutic option for IIH, since obesity is one of the leading causes of IIH. The mechanism of how topiramate leads to weight loss is not fully understood, but it is thought to be related to decreased calorie intake due to increased hypothalamic cortisol-releasing hormone, which inhibits feeding behavior [13]. Topiramate can also improve papilledema, which is due to increased ICP, and has always been used in therapy for primary headaches, such as migraine prevention [12,14]. The side effects of topiramate include ophthalmic manifestations such as ciliochoroidal effusion syndrome, deterioration in neurocognitive function like language comprehension, and working memory [15,16]. Also, paresthesia, fatigue, dizziness, and ataxia have been observed in higher doses of topiramate. Therefore, monitoring of the side effects must be ensured especially with the escalation of therapeutic dosage [17].

An animal trial investigated the effect of using five drugs with diuretic effects in IIH (acetazolamide, topiramate, furosemide, amiloride, and octreotide), which were injected subcutaneously at clinical doses. Topiramate showed the most favorable effect. Topiramate decreased the ICP significantly over 2 h to 68.6  ±  2.0% of baseline (32% reduction, *p*  <  0.001). Acetazolamide showed ICP reduction activity, but not as significantly as when compared to the control group [18].

To date, there are few human clinical trials and few cohorts comparing acetazolamide and topiramate separately. In an open-label study, the clinical outcomes of 40 patients diagnosed with IIH were assessed and then compared between the two treatment groups. Both topiramate and acetazolamide had a remarkable effect in decreasing ICP, in the retrieval of visual function, in resolving headaches, and in the disappearance of transient visual obscuration and intracranial noises. Nevertheless, when both treatment groups were compared, no statistically significant difference was found between them in treating IIH [10].

Significant gaps remain in understanding the most effective therapeutic agent, the optimal long-term management strategies, and the impact on quality of life when using acetazolamide and/or topiramate to treat idiopathic intracranial hypertension (IIH). Addressing these gaps could improve management strategies. Our aim in this study is to explore the clinical outcomes of topiramate and acetazolamide in the treatment of IIH, evaluate their long-term outcomes, and recommend the best therapeutic option for IIH.

## 2. Materials and Methods

### 2.1. Guidelines and Protocol Registration

This review followed the Preferred Reporting Items for Systematic Reviews and Meta-Analyses (PRISMA) guidelines. Prior to starting, the review protocol, identified by ID CRD42024529867, was submitted to and approved by the International Prospective Register for Systematic Reviews (PROSPERO).

### 2.2. Search Scheme

An electronic literature search was conducted from 7 June 2024 to 19 October 2024 across several databases, including MEDLINE, EMBASE, PubMed, Scopus, and Web of Science. The aim was to identify studies that compared the efficacy of topiramate and acetazolamide as medical treatments for IIH and assessed the long-term outcomes of both medications. Controlled search vocabularies (MeSH) were applied, combining the following search terms: (“topiramate” OR “acetazolamide”) AND (“Idiopathic intracranial hypertension” OR “IIH” OR “pseudotumor cerebri syndrome”) (Supplementary File S1, Table S1).

### 2.3. Eligibility Criteria

This research included randomized clinical trials (RCTs) as well as retrospective and prospective cohort studies that specifically examined the effects of either acetazolamide or topiramate on patients diagnosed with idiopathic intracranial hypertension (IIH) who met the modified Dandy criteria, with no restrictions based on age, gender, or race. The outcomes assessed were reductions in intracranial pressure, headache relief, and changes in body mass index (BMI). The Dandy criteria required the presence of signs and symptoms of increased intracranial pressure (e.g., papilledema, headache), normal neuroimaging, normal cerebrospinal fluid composition, and elevated cerebrospinal fluid opening pressure, in the absence of other identifiable causes of intracranial hypertension.

### 2.4. Data Extraction (Selection and Coding)

The Rayyan application (Qatar Computing Research Institute, Ar-Rayyan, Qatar) was used to facilitate the screening and selection of relevant articles for inclusion. Two reviewers (RH and AA) independently reviewed the abstracts and titles of the articles to identify potentially relevant studies, with any disagreements resolved through discussion and consensus. The data extracted from each study included the authors, study design, inclusion and exclusion criteria, country of origin, quality assessment, sample size, demographic and intervention details, outcome measures, and conclusions. In addition, the completed PRISMA checklist is provided as a supplementary file (Supplementary File S1, Table S2).

### 2.5. Risk of Bias (Quality) Assessment

Two reviewers independently conducted risk of bias assessments using the Critical Appraisal Skills Program (CASP) tool, which offers different versions based on the study design. Any disagreements were resolved through discussion with the three other collaborators involved in the study.

### 2.6. Strategy for Data Synthesis and Analysis

A comprehensive search was conducted across various databases to identify relevant research. To address statistical heterogeneity, a random-effects model was employed. The key parameters from each study were extracted and organized into tables. The available data were carefully discussed to determine the most suitable statistical methods for analysis.

The meta-analysis was performed using the R program. The heterogeneity between studies was evaluated using the Cochran’s Q and I2 tests. A fixed-effects model was used for meta-analysis where heterogeneity was less than 50%. When heterogeneity was greater than 50%, a random-effects model was used.

## 3. Results

**Figure 1:**
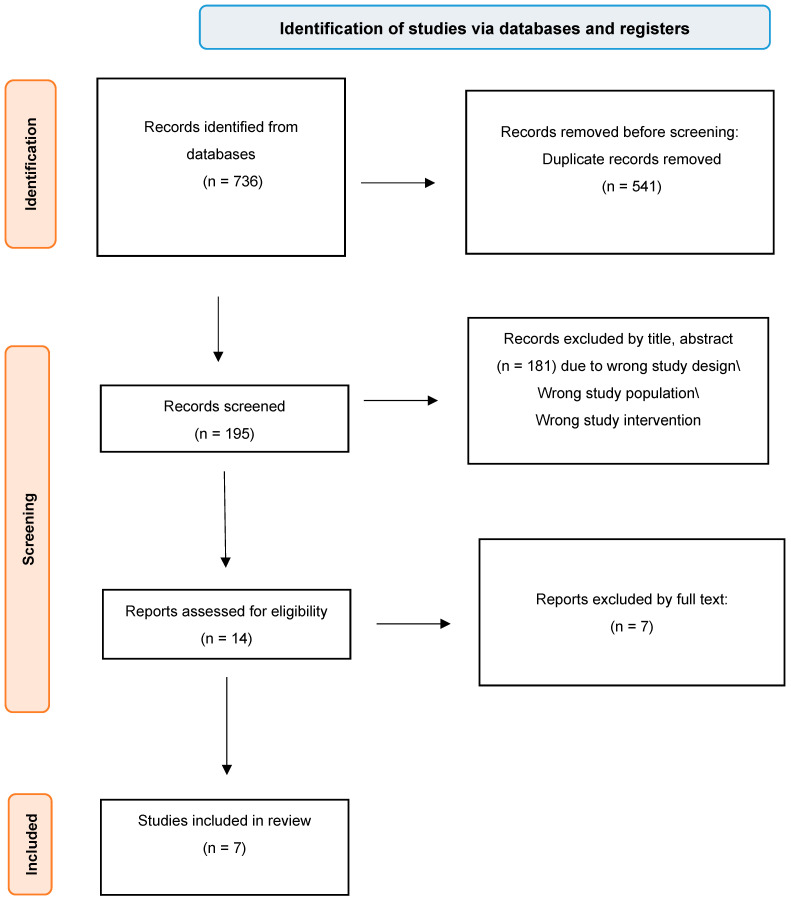
PRISMA flow diagram.

Our search identified a total of 736 studies, of which 541 were duplicates and 181 were excluded after screening the abstracts and titles. The full texts of the remaining 14 records were obtained to assess eligibility. After a comprehensive examination, seven studies were found to be appropriate for selection, as illustrated in Figure 1.

Table 1 provides a comprehensive overview of the characteristics of the studies included in the analysis, focusing on idiopathic intracranial hypertension (IIH).

All trials, except for study by Togha et al. (2020) [19], which was retrospective, and the study by Celebisoy et al. (2007) [10], which was an open-label clinical trial, were prospective cohort studies. These studies were conducted in various countries, including Iran, Turkey, Denmark, Italy, India, and the United Kingdom.

The studies included in this systematic review exhibited significant variation in sample sizes, ranging from larger cohorts such as Thaller et al. (2023) [20] with 490 participants, to smaller cohorts like Lochner et al. (2018) [21], which included only 22 individuals.

In the included studies, the follow-up durations ranged from as short as six months to nearly seven years. Thaller et al.’s (2023) [20] study had a mean follow-up time of 23.5 months, with a range of 0 to 87 months [20]. This variation in follow-up duration may influence the results and conclusions.

While most studies focus on a single cohort of IIH patients, there are exceptions. For example, Togha et al. [19] included four treatment groups and investigated the outcomes of recurrent lumbar punctures, combination therapy, acetazolamide, and surgery. The quality assessment results indicated that three studies were of high quality [20,21,22], while four studies were of moderate quality [10,19,23,24].

**Table 1:** Characteristics of the included studies.

Study ID	Country	Study Design	Sample Size	Duration of Follow-Up	Study Groups	Quality Assessment
Azharudeen, 2023 [23]	India	Prospective cohort	49	6 months	One group (not mentioned)	Moderate quality
Lochner, 2018 [21]	Italy	Prospective cohort	22	6 months	One group with acetazolamide	High quality
Takkar, 2018 [24]	India	Prospective cohort	40	6 months	One group with topiramate and acetazolamide	Moderate quality
Thaller, 2023 [20]	United Kingdom	Prospective cohort	490	Mean duration of follow-up of 23.5 (range of 0–87) months.	Medically and surgically managed with acetazolamide, topiramate, and other diuretics	High quality
Yri, 2014 [22]	Denmark	Prospective cohort	44	12 months	One group with acetazolamide and/or topiramate	High quality
N Celebisoy, 2007 [10]	Turkey	Open-label clinical trial	40	12 months	Two groups with acetazolamide	Moderate quality
Togha, 2020 [19]	Iran	Retrospective cohort	202	NA	Four groups with acetazolamide (group 1), acetazolamide plus topiramate or Lasix (group 2), repeated LP (group 3), and surgical intervention (group 4)	Moderate quality

### 3.1. Characteristics of the Studies’ Participants

A detailed summary of the participant characteristics in the included studies is presented in Table 2.

#### 3.1.1. Number of Participants and Distribution of the Groups

The included studies showed considerable variation in participant numbers. For instance, ref. [21] included just 22 participants, while [20] had a much larger cohort of 490. To facilitate a focused evaluation of therapies, some studies, such as [10], specifically divided participants into treatment groups, such as acetazolamide versus topiramate.

#### 3.1.2. Distribution of Ages

The age distribution of participants in the included studies aligned with the typical demographic pattern of IIH, which predominantly affects young- to middle-aged individuals. For instance, ref. [23] had a mean age of 29.8 years, refs. [19,21] reported mean ages of 33 ± 11 and 33.17 years, respectively.

#### 3.1.3. Representation of Gender

The results of this study showed that the majority of participants across all studies were women. For instance, ref. [20]) reported 481 females and only 9 males, while [23] had 98% female participation. This finding was consistent with the well-established higher incidence of IIH in women, especially people with obesity.

#### 3.1.4. Criteria for Inclusion and Exclusion

All studies included in this systematic review established clear inclusion criteria, ensuring that participants met diagnostic standards such as the modified Dandy criteria or other recognized protocols for IIH. The exclusion criteria were similarly specific. For example, ref. [20] excluded individuals with secondary causes of intracranial hypertension, IIH without papilledema (IIHWOP), or unconfirmed diagnoses. This focus on confirmed IIH cases strengthened the validity of the data. Additionally, notable exclusions in other studies included patients with clinically evident optic nerve atrophy, in [22], or secondary causes of increased intracranial pressure, in [19].

#### 3.1.5. Diagnosed Disease

In most of the studies included in this systematic review, the modified Dandy criteria or other accepted diagnostic standards were used to diagnose patients, ensuring consistency in the inclusion criteria. Togha et al. [19] relied on the European Headache Federation’s standards for diagnosis, which involved excluding secondary causes and identifying signs and symptoms of elevated intracranial pressure.

#### 3.1.6. Descriptions of Treatment

Topiramate: The recommended daily dosage of topiramate varies significantly across the included studies, ranging from 25 mg [24] to 400 mg [20]. A personalized approach, involving the stepwise titration of the dosage based on patient response and tolerance, was recommended by [10].

Acetazolamide is a common therapy for IIH, and acetazolamide was administered in varying dosages across the studies, ranging from 250 mg to 4 g per day [20]. This dosage variation reflected patient tolerances and differing clinical practices (Table 3).

#### 3.1.7. Principal Results

The primary outcomes in all of the studies included in this systematic review were centered on neurological and visual parameters. Visual outcomes were evaluated using metrics such as peripapillary retinal nerve fiber layer (RNFL) thickness, optic disc elevation, and visual acuity, e.g., [20].Furthermore, cerebrospinal fluid (CSF) pressure measurements were employed to assess treatment effectiveness and confirm the presence of elevated intracranial pressure.

Papilledema: Grading systems, such as Frisen’s scale, were commonly used to assess the severity of papilledema [10].

### 3.2. Secondary Outcomes

Broader health indicators were considered secondary outcomes in the research. Headache severity: Most studies included in this research (e.g., [22,23]) used measures such as the Visual Analog Scale (VAS) or the Numerical Rating Scale (NRS) to assess the frequency and severity of headaches. Changes in body mass index (BMI) were identified as an important modifiable factor during the course of IIH (e.g., [20]). Imaging techniques: MRI and optical coherence tomography (OCT) were used to reveal the structural changes associated with IIH (Table 3).

### 3.3. Measures of the Outcomes

Standardized instruments including lumbar puncture manometry, gadolinium-enhanced MRI, and Humphrey visual field tests are commonly used. According to [21], longitudinal imaging supports therapy assessment by integrating objective ocular ultrasonography data with clinical observations.

In summary, topiramate’s effectiveness in treating IIH symptoms is demonstrated by several studies, and it also aids patients in losing weight [10]. It is a useful therapy choice because of its dual mode of action, which lowers CSF development and promotes weight reduction.

### 3.4. Long-Term Management Challenges

Studies like [22] have shown persistent headaches, even after papilledema has resolved, indicating that the processes behind headaches in IIH go beyond an increase in intracranial pressure.

Factors such as BMI changes, baseline visual status, and disease duration are all frequently recognized as important prognostic indicators. The focus is on early diagnosis and intervention [20,24]. The main clinical concerns in IIH management are shown in the research’s alignment in focusing on important outcomes such as visual metrics, CSF pressure, and headache intensity.

Variability in research designs: Direct comparisons are difficult due to the variations in group formations, follow-up times, and treatment doses. These differences do, however, offer a wide range of information for comprehending IIH treatment.

### 3.5. Benefits and Drawbacks of Treatment Methods

Given that obesity is a controllable risk factor, topiramate’s added benefit of weight loss is a major plus in IIH. However, because of its proven effectiveness, acetazolamide continues to be the standard therapy.

#### 3.5.1. Unresolved Issues

Even when other symptoms have subsided, persistent headaches indicate the need for more investigation into the pathophysiology of headaches associated with IIH and possible complementary treatments.

#### 3.5.2. Controlling Weight

Numerous research suggests that weight should be controlled, as it is essential in achieving better results [10,20]. This emphasizes how crucial it is to incorporate lifestyle modifications into IIH treatment plans; in particular, weight loss, dietary modifications, and physical activity.

### 3.6. Quality Assessment of the Included Studies

**Figure 2:**
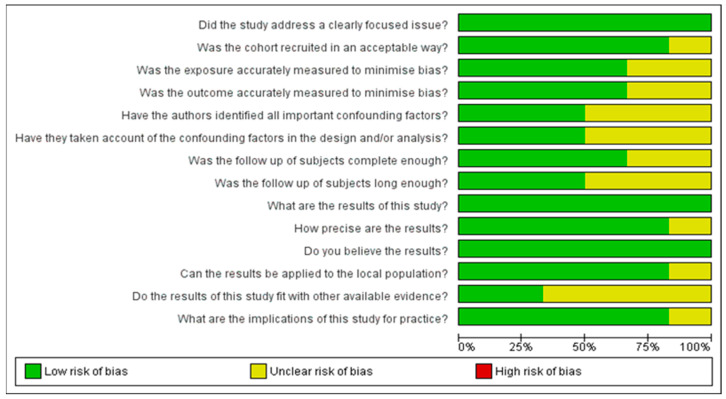
Risk of bias graph.

**Figure 3:**
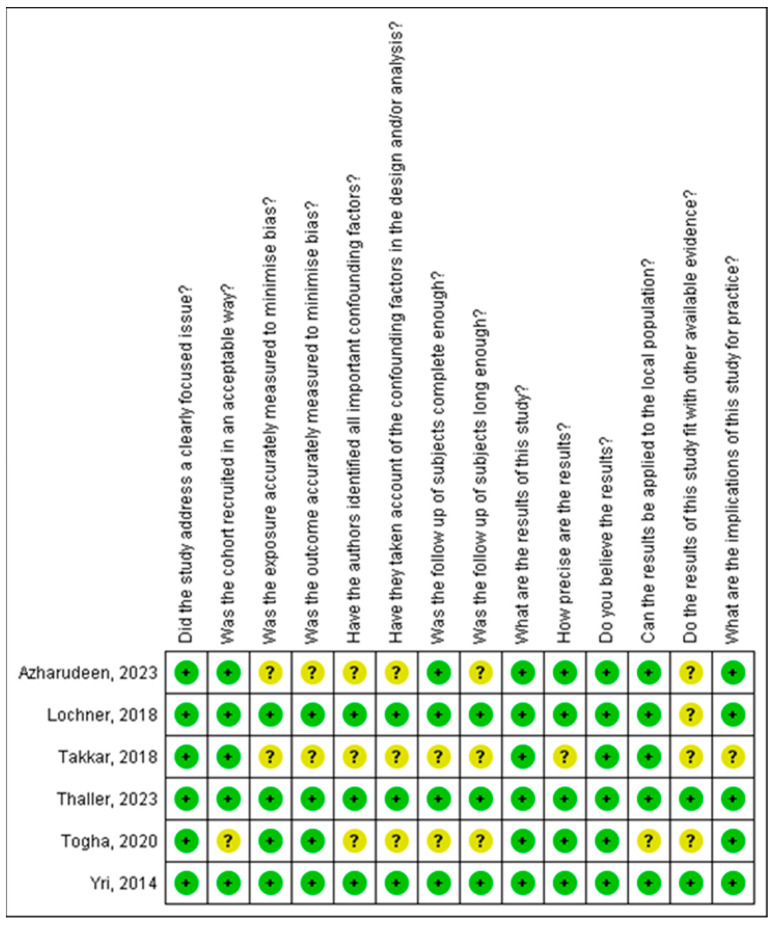
Risk of bias summary [19,20,21,22,23,24].

Figure 2 and Figure 3 present a thorough analysis of potential bias in the studies included in this systematic review. While Figure 2 provides detailed information for each study, Figure 3 offers an overview of the main patterns. In addition to highlighting strengths, such as accurate outcome assessments, the figure also identifies limitations, including the inadequate control of confounding factors and insufficient follow-up.

### 3.7. Meta-Analysis

#### 3.7.1. Headache Severity at 3 Months

**Figure 4:**
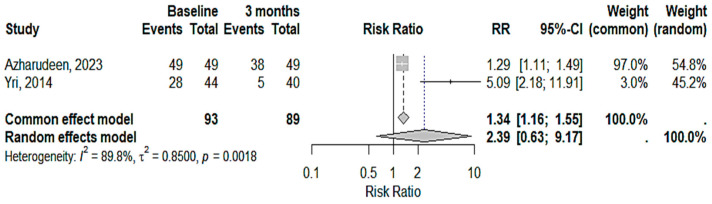
Improvement in headache severity in IIH patients with acetazolamide and topiramate after 3 months [22,23].

The results revealed a reduction in headache severity after three months, with a confidence interval not exceeding one (Figure 4). The fixed-effects model predicted a significant decrease in headache severity after three months, while the random-effects model showed no significant differences. The heterogeneity among the studies was statistically significant, indicating substantial variations in the research outcomes. Although the fixed-effects model produced consistent results, the high variability warranted caution when generalizing these findings

#### 3.7.2. Headache Severity at 6 Months

**Figure 5:**
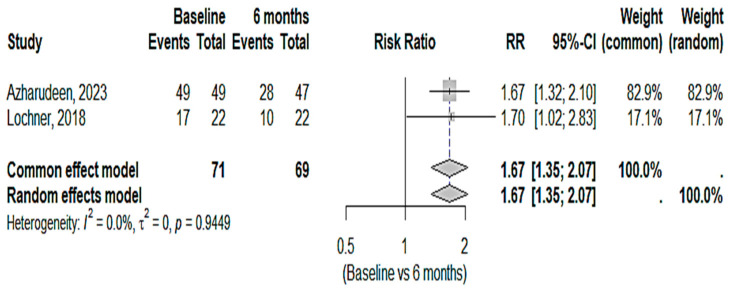
Improvement in headache severity in IIH patients with acetazolamide and topiramate after 6 months [21,23].

The findings revealed a statistically significant 67% increase in the likelihood of improvement after 6 months compared to the baseline (Figure 5). Both studies demonstrated consistent results with no substantial heterogeneity. The risk ratio (RR) indicated a significant reduction in intensity at 6 months, with fewer subjects experiencing headaches. The data pointed to a statistically significant and consistent improvement in headache severity at 6 months compared to the baseline, with no heterogeneity and uniform outcomes across both studies. This demonstrated that the intervention led to a significant reduction in headache severity over time.

#### 3.7.3. Visual Obscuration Reduction

**Figure 6:**
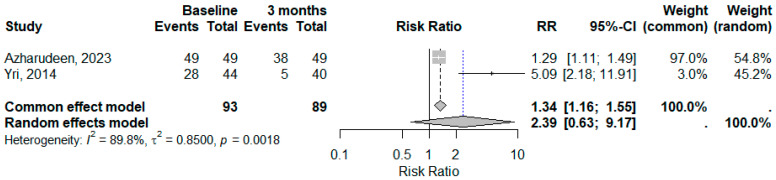
Improvement in visual obscuration in IIH patients with acetazolamide and topiramate after 3 months [22,23].

The meta-analysis of visual obscuration events between the baseline and six months revealed a significant decrease in risk. The pooled RR for the fixed-effects model was 3.57, while for the random-effects model it was 3.60. Both studies demonstrated low heterogeneity, with no substantial variability. The findings indicated that patients at the baseline were 3.6 times more likely to experience visual obscuration compared to those at six months (Figure 6).

#### 3.7.4. Visual Analog Scale (VAS) Reduction

**Figure 7:**
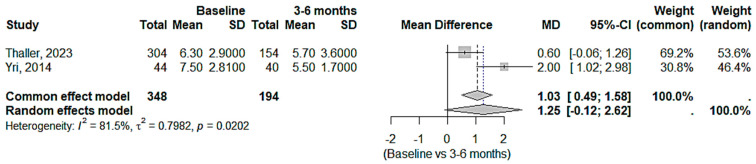
VAS reduction in IIH patients with acetazolamide and topiramate after 3–6 months [20,22].

The pooled findings from the fixed-effects model indicated a non-significant reduction in VAS scores at 3–6 months compared to the baseline (MD = 1.03, 95% confidence interval: 0.49 to 1.58). The random-effects model accounted for considerable heterogeneity (I^2^ = 81.5%) but did not show a statistically significant improvement (MD = 1.25, 95% CI: −0.12 to 2.62). While there was some reduction in pain levels after 3–6 months, the great heterogeneity and non-significant random-effects results indicated that the effectiveness of the medications varied among trials (Figure 7).

## 4. Discussion

According to the current study, using acetazolamide and topiramate at 6 months reduced headache severity by 67% when compared to the baseline. Given the similar findings from both studies and the lack of significant heterogeneity, it appears that these medications are useful in gradually reducing the intensity of headaches. In clinical studies for idiopathic intracranial hypertension (IIH), acetazolamide, a carbonic anhydrase inhibitor, has been shown to lower intracranial pressure and alleviate headache symptoms [4,25]. A meta-analysis of topiramate’s effectiveness in managing headache revealed that, when compared to a placebo, it significantly reduced the number of headache days [26]. For the treatment of headaches, combination therapy has also been investigated; certain evidence indicates that the combination may be more beneficial than each medication alone, especially for individuals with refractory headaches [27].

According to the current meta-analysis, the probability of visual obscuration occurrences during a six-month period significantly decreased, with a relative risk (RR) of 3.60 for the random-effects model and 3.57 for the common effects model. The trustworthiness of the findings is strengthened by the minimal heterogeneity shown in both models, which suggests that the outcomes are consistent throughout the included studies. Acetazolamide and a low-sodium weight-reduction diet were compared to dieting alone in a prior trial when it came to individuals with IIH and who had minor vision loss [11]. Visual field function improved moderately with the combination therapy according to the results. Comprehensive information on the diagnosis and treatment of IIH is provided by the Recommendations on the Diagnosis and Management of IIH. Acetazolamide and topiramate are used to lower intracranial pressure and enhance visual results [28].

IIH is treated with acetazolamide and topiramate [29,30]. Acetazolamide lowers intracranial pressure by decreasing the production of cerebrospinal fluid (CSF) [3,31,32]. It is regarded as the initial course of therapy for IIH. Correspondingly, topiramate effectively lowers intracranial pressure and headache symptoms while simultaneously inhibiting carbonic anhydrase [33,34]. Additionally, it encourages weight loss, which is advantageous for people with IIH who are overweight. Over the course of three to twelve months, it has been demonstrated that both medications considerably enhance visual fields in IIH patients [35,36].

However, tiredness, paresthesia, and gastrointestinal disturbances are frequent adverse effects of both medications [37,38]. According to a study comparing the two medications, topiramate is effective and well tolerated in treating IIH, making it an acceptable alternative for acetazolamide, especially in patients who are class III obesity, due to its ability to reduce body weight [10]. The main limitation of using topiramate to treat IIH is its expensive cost, as well as its adverse effects like myopia and acute angle-closure glaucoma [39,40].

In summary, topiramate and acetazolamide both are effective in treating IIH, and they are equally effective in lowering intracranial pressure and enhancing visual fields. However, each patient’s reaction and tolerance to side effects may influence the pharmaceutical selection. It is difficult to generalize the findings of this study due to several limitations, such as heterogeneity, inconsistent results, and inadequate long-term data.

## 5. Conclusions

According to this study, acetazolamide and topiramate are useful therapies for idiopathic intracranial hypertension (IIH). They exhibit improvements in visual metrics and decreased cerebrospinal fluid (CSF) pressure. After six months, the intensity of the headaches significantly improved with both therapies. Persistent headaches and variations in certain results lead to the need for more investigations and customized interventions. One important modifiable aspect of the therapy for IIH is weight reduction, which topiramate helps with. Because it effectively lowers CSF pressure and alleviates visual problems, acetazolamide is still the recommended course of therapy. For best results, topiramate and acetazolamide combination treatment is recommended. In addition to medication, lifestyle changes including diet and physical activity are crucial, and treatment monitoring is essential. Early diagnosis and treatments are advisable, along with dose modification according to the condition of the patient. Additionally, further clinical trials and cohort studies are necessary to compare the effectiveness of the two treatments topiramate and acetazolamide over longer periods.

## Figures and Tables

**Table 2:** Characteristics of the studies’ participants.

Study ID	Number of Participants in Each Group	Age	Inclusion Criteria	Exclusion Criteria	Disease Diagnosis
Azharudeen, 2023 [23]	49 (females (98%))	Ranging from 16 to 62 years, mean ± SD age of the cases was 29.8 ± 9.7 years	Patients fulfilling the modified Dandy criteria for IIH were recruited	Not mentioned	Patients fulfilling the modified Dandy criteria for IIH
Lochner, 2018 [21]	22 (16 females and 6 males)	Mean age of 33 ± 11 years	Not mentioned	Not mentioned	Patients diagnosed with pseudotumor cerebri syndrome according to the current diagnostic criteria
Takkar, 2018 [24]	40 (38 females and 2 males)	Mean age of 32.8 ± 11.06 years (range 14–56 years)	All consecutive patients fulfilling the modified Dandy criteria	Not mentioned	The modified Dandy criteria for IIH
Thaller, 2023 [20]	426 medically managed, 64 surgically managed (481 females, 9 males)	Mean of 31.5 years (9.4)	A confirmed diagnosis of IIH as per the modified Dandy criteria	A secondary cause of intracranial hypertension, and those with IIH without papilledema	Modified Dandy criteria
Yri, 2014 [22]	44 (43 females and 1 male)	Median (IQR): 27.5 years (22.3–35.3)	Only patients with new-onset IIH referred before or within 7 days of diagnostic lumbar puncture	Patients with clinically detectable optic nerve atrophy	Not mentioned
N Celebisoy, 2007 [10]	20 participants in each group. Acetazolamide M/F: 3/17; topiramate M/F: 2/18	Acetazolamide group median: 35 years (16–49). Topiramate group: 32 (16–51)	Not mentioned	Not mentioned	Snellen visual acuities, ophthalmoscopy, and automated perimetry
Togha, 2020 [19]	Group 1: 67, Group 2: 23, Group 3: 81, Group 4: 31 patients (females (78%))	Mean age of 33.17 years	Patients aged 16 to 60 who fulfilled the diagnosis criteria of IIH based on the European Headache Federation guidelines	Not mentioned	Not mentioned

**Table 3:** Description of treatment medications and outcomes.

Study ID	Description of Low Dose Topiramate	Description of Acetazolamide	Primary Outcomes	Secondary Outcomes	Outcome Measurement	Conclusion
Azharudeen, 2023 [23]	Not mentioned	Not mentioned	Grades of papilledema and CSF opening pressure	Headache severity	• Measured cerebrospinal fluid opening pressure in the lateral decubitus position. • Numerical Rating Scale used for headache assessment.	• Good vision prognosis. • Suspected in obese women with headaches. • Preventable cause of blindness.
Lochner, 2018 [21]	Median dose 150 mg/day, range of 100–200	Acetazolamide (mean dose 500 mg/day, range of 250–3000)	Optic nerve sheath diameter and optic disc elevation	Headache intensity, BMI	• Brain MRI and CT scans. • Visual Analog Scale for headache intensity.	Follow-up with ocular ultrasound combined with clinical information may provide support for the treatment of this condition.
Takkar, 2018 [24]	Topiramate 25 mg to 100 mg	Acetazolamide 500 mg to 2 mg	Humphrey mean deviation, visual acuity, and CSF opening pressure	Retinal nerve fiber layer (RNFL) thickness	Contrast Sensitivity Measurements in patients: • Functional acuity contrast test; • Gadolinium-enhanced MRI.	• Visual loss at presentation is a key predictor of final outcome. • Early diagnosis and prompt management are key.
Thaller, 2023 [20]	Mean dose 135 mg/day, range of 25 to 400 mg daily	Mean dose 1032 mg/day, range of 250 mg to 4 g daily	Visual acuity and Humphrey visual field and optical coherence tomography imaging measurements	Headache frequency, severity	Optical coherence tomography imaging, parameters of average global peripapillary retinal nerve fiber layer.	Severe papilledema visual outcomes: • Delayed decline in visual outcomes. • Disease duration and BMI changes are key prognostic factors.
Yri, 2014 [22]	Range of 100–125 mg/day	Range of 750–2225 mg/day with acetazolamide and/or topiramate	Headache and ICP	BMI	Optical coherence tomography, standardized lumbar puncture intracranial pressure manometry.	• A total of 43% of patients responded well to ICP management. • Remaining patients experienced sustained long-term headaches despite papilledema resolution. • High ICP and young age linked to better headache outcomes.
N Celebisoy, 2007 [10]	Range of 100 to 150 mg daily, started at a dose of 50 mg /day and gradually titrated to the efficacious dose	Range of 1000 to 1500 mg, a titration schedule that started with 500 mg/day was used for acetazolamide	Cerebrospinal fluid pressure and visual field grades	BMI	MRI, and the degree of papilledema was graded by using Frisen’s scheme.	Topiramate appears to be effective in treating IIH due to its mechanism of action, which involves weight reduction and decreased CSF formation.
Togha, 2020 [19]	Not mentioned	Not mentioned	Headache, disease severity	BMI	CSF pressure, MRI, and LP results.	• Headache is the most common symptom, especially in the acetazolamide group. • Blurred vision is the second most common symptom, and is more common in the surgery group. • Higher CSF pressure may indicate poor therapy response and the need for intensive treatment. • Headache without blurred vision may indicate mild severity and good treatment response.

## Data Availability

All relevant data supporting the findings of this study are included within the manuscript.

## Supplementary Materials

The following supporting information can be downloaded at: https://www.mdpi.com/article/10.3390/medicina61030450/s1, Table S1. Search strategy used in different databases. Table S2. Preferred Reporting Items for Systematic Reviews and Meta-Analyses (PRISMA) checklist.
